# Genomic and clinical epidemiology of SARS-CoV-2 in coastal Kenya: insights into variant circulation, reinfection, and multiple lineage importations during a post-pandemic wave

**DOI:** 10.1186/s44263-025-00201-6

**Published:** 2025-09-09

**Authors:** Arnold W. Lambisia, Esther N. Katama, Edidah Moraa, John M. Mwita, Katherine Gallagher, Martin Mutunga, Emily Nyale, Joan Omungala, Mike Mwanga, Nickson Murunga, Joyce Nyiro, James Nyagwange, Charles Sande, Philip Bejon, George Githinji, Simon Dellicour, My V. T. Phan, Matthew Cotten, L. Isabella Ochola-Oyier, Edward C. Holmes, Charles N. Agoti

**Affiliations:** 1 Kenya Medical Research Institute (KEMRI) - Wellcome Trust Research Programme (KWTRP), Kilifi, Kenya; 2 Department of Infectious Diseases Epidemiology, London, School of Hygiene and Tropical Medicine , Keppel Street, London, UK; 3 University of Oxford, Oxford, UK; 4 Pwani University, Kilifi, Kenya; 5 Spatial Epidemiology Lab (SpELL), Université Libre de Bruxelles, Brussels, Belgium; 6 Department of Microbiology, Immunology and Transplantation, Rega Institute & Laboratory for Clinical and Epidemiological Virology, University of Leuven, Louvain, KULeuven, Belgium; 7 College of Health Solutions, Arizona State University, Phoenix, AZ USA; 8 Complex Adaptive System Initiative, Arizona State University, Scottsdale, AZ USA; 9 School of Medical Sciences, University of Sydney, Sydney, Australia

**Keywords:** COVID-19, SARS-CoV-2, Omicron, XBB.2.3, JN.1, Reinfections, Coastal Kenya

## Abstract

**Background:**

Between November 2023 and March 2024, coastal Kenya experienced another wave of severe acute respiratory syndrome coronavirus 2 (SARS-CoV-2) infections detected through our continued genomic surveillance. Herein, we report the clinical and genomic epidemiology of SARS-CoV-2 infections from 179 individuals (a total of 185 positive samples) residing in the Kilifi Health and Demographic Surveillance System (KHDSS) area (~ 900 km^2^).

**Methods:**

We analyzed genetic, clinical, and epidemiological data from SARS-CoV-2 positive cases across pediatric inpatient, health facility outpatient, and homestead community surveillance platforms. Phylogenetic analyses were performed using maximum-likelihood and Bayesian frameworks. Temporal trends were summarized, comparisons conducted using Kruskal–Wallis and Wilcoxon tests, and associations examined using univariate and multivariable logistic regression models.

**Results:**

Sixteen SARS-CoV-2 lineages within 3 subvariants [XBB.2.3-like (58.4%), JN.1-like (40.5%), and XBB.1-like (1.1%)] were identified. The symptomatic infection rate was estimated at 16.0% (95% CI, 11.1–23.9%) based on community testing regardless of symptom status and did not differ across the subvariants (*p* = 0.13). The most common infection symptoms in community cases were cough (49.2%), fever (27.0%), sore throat (7.3%), headache (6.9%), and difficulty in breathing (5.5%). One case succumbed to the infection. Genomic analysis of the virus from serial positive samples confirmed repeat infections among 5 participants under follow-up (median interval 21 days, range 16–95 days); in 4 participants, the same virus lineage was responsible in both episodes, whereas 1 participant had a different lineage in the second compared with the first episode. Phylogenetic analysis including > 18,000 contemporaneous global sequences provided evidence for at least 38 independent virus introduction events into the study area (KHDSS) during the wave, the majority likely originating in North America and Europe.

**Conclusions:**

Our study highlights that coastal Kenya, like most other localities, continues to face new SARS-CoV-2 infection waves characterized by circulation of new variants, multiple lineage importations, and reinfections. Locally, the virus may circulate unrecognized, as most infections are asymptomatic in part due to high population immunity after several waves of infection. Our findings highlight the need for sustained SARS-CoV-2 surveillance to inform appropriate public health responses, such as scheduled vaccination for populations at risk of severe infection.

**Supplementary Information:**

The online version contains supplementary material available at 10.1186/s44263-025-00201-6.

## Background

The coronavirus disease 2019 (COVID-19) pandemic (2020–2023) witnessed globally unprecedented levels of virus genomic surveillance with > 14 million partial or complete genomes of SARS-CoV-2 (the causative agent of COVID-19) sequenced [1]. This enabled detailed investigation into the molecular epidemiology of SARS-CoV-2, in turn revealing the emergence of variants of concern (VOC), modes of evolution (including single-nucleotide polymorphisms (SNPs), recombination, sequence deletions), and the pathways of virus spread across different scales of observation, information that together contributed to the rational design and key updates of control tools including vaccine strain composition for booster doses [2].

When the World Health Organization (WHO) declared an end to the public health emergency of international concern status (PHEIC) of COVID-19 in May 2023 [3], SARS-CoV-2 genomic surveillance substantially decreased, despite its clearly demonstrated value [4, 5]. This has led to a paucity of systematically collected genomic and epidemiological data for recently emerged SARS-CoV-2 variants/lineages such as JN.1, XBB.2.3, LF.8, and XEC, especially in low- and middle-income country (LMIC) settings. In the meantime, waves of SARS-CoV-2 infections continue to appear, particularly driven by novel variants that may exhibit increased transmissibility, virulence, immune escape, or any combination of these characteristics [6].

Kenya, a lower-middle-income country in East Africa with a population of ~ 50 million people, recorded its first COVID-19 case on March 13, 2020 [7]. COVID-19 vaccination started in the country in March 2021, and as of November 2022, approximately 11.1% of the Kenyan population had received ≥ 1 dose of the COVID-19 vaccine [8]. Since March 2020, the country has consistently recorded 1–3 waves of SARS-CoV-2 infections annually, with the coastal region of Kenya reporting 7 waves of SARS-CoV-2 infection by the end of May 2023 [9–12]. The waves were dominated by B.1* (wave peak in July 2020), B.1* (wave peak in November 2020) [12], B.1.1.7 (Alpha VOC) and B.1.351 (Beta VOC; March–April 2021), B.1.617.2* (Delta VOC; July 2021) [13], BA.1* (Omicron VOC; January 2022), BA.4/5* (Omicron; June 2022), BQ.1* (Omicron; November 2022), and FY.4* (Omicron, alias XBB.1.22.1.4; April 2023) [14].

While there is substantially reduced SARS-CoV-2 genomic surveillance in Kenya, continuous genomic surveillance has generated at least 13,816 SARS-CoV-2 partial or complete genome sequences available in the Global Initiative on Sharing All Influenza Data (GISAID) database as of October 6, 2024, enabling a comprehensive tracking of the pandemic at a national scale [10–12, 15, 16]. By the end of 2022, more than 70% of Kilifi residents (a rural population in coastal Kenya) and 90% of Nairobi residents (an urban population in Kenya’s capital city) were estimated to be seropositive for SARS-CoV-2 anti-S IgG [8]. Despite this apparent high infection rate, Kenya, like most other sub-Saharan African countries, experienced a relatively mild COVID-19 disease burden, with > 80% of the infections asymptomatic. However, data to support this conclusion are limited to a few community studies [17, 18]. As new waves of infection appear in the region, genomic surveillance remains an important tool to identify the variants involved, study evolution, and reveal their impact on current control measures such as vaccines.

**Fig. 1:**
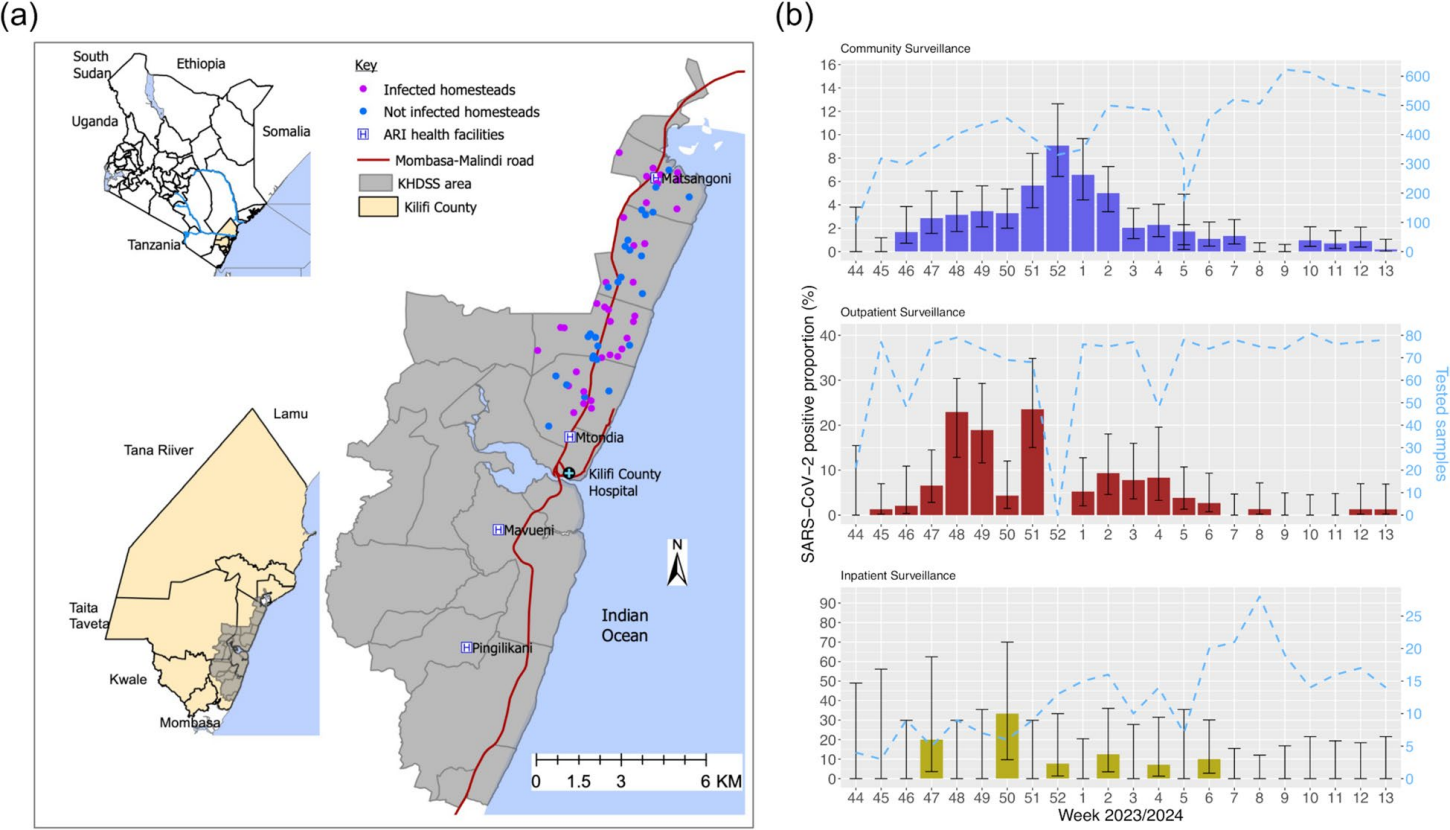
Study population and surveillance platforms. **a** A map of the study area showing Kilifi Health and Demographic Surveillance System (KHDSS), Kilifi, Kenya (the large map), and its location within Kilifi County and Kenya. The pink and blue dots show the distribution of the recruited homesteads in the community surveillance arm. The location of the 5 health facilities for inpatient (KCH) and/or outpatient surveillance is indicated. The map was generated using ArcGIS software. **b** Temporal trends of SARS-CoV-2 positive samples from October 2023 to March 2024 in Kilifi, Kenya. Specifically, we report weekly SARS-CoV-2 detection in the KHDSS area between October 2023 and March 2024 across the 3 surveillance platforms: community, outpatient, and inpatient. The dashed line shows the total number of samples tested per week (secondary *y*-axis) across the different platforms

Within coastal Kenya, the KEMRI-Wellcome Trust Research Programme (KWTRP) runs the Kilifi Health and Demographic Surveillance System area (KHDSS) [19]. In the KHDSS area, KWTRP currently runs 3 surveillance platforms for monitoring respiratory disease epidemiology: community (weekly homestead visits) surveillance, outpatient (5 health facilities) surveillance, and inpatient paediatric (< 5-year-olds) pneumonia surveillance (Kilifi County Referral Hospital (KCRH), Fig. 1) [20, 21]. The patterns of repeat infections during new community waves of SARS-CoV-2 transmission have not been reported anywhere in Africa outside of Tunisia and South Africa [18, 22]. Here, we describe the epidemiological patterns from the analyses of SARS-CoV-2 genomic data collected at the KHDSS during the wave of SARS-CoV-2 infections that occurred between November 2023 and March 2024 in coastal Kenya (8th wave).

## Methods

### Study design and population

This study was conducted within the KHDSS, in the Kilifi County of coastal Kenya (Fig. 1). The study participants were primarily residents of the KHDSS [19]. The KHDSS area covers ~ 900 km [2], has a population size ~ 300,000 people of diverse ethnic groups, with the predominant communities being Mijikenda-speaking people [19]. The population is characterized by a youthful demographic profile with 62% of the population below 30 years and includes both rural and urban settings [23].

Respiratory specimens were collected from individuals recruited across 3 respiratory disease surveillance platforms established by the Pathogen Epidemiology and Omics (PEO) Group within KWTRP. The platforms include the following:

- Pediatric inpatient (IP) pneumonia surveillance: since 2002, respiratory samples (nasal washing, nasopharyngeal aspirates, or NP/OP swabs) have been collected from children aged < 5 years old admitted to KCRH presenting with symptoms defined as severe or very severe pneumonia according to the WHO definition [24]. Note that although KCRH serves both residents and non-residents of KHDSS, most patients seeking care at KCRH are KHDSS residents.
- Health facility outpatient (OP) surveillance: since December 2020, NP/OP swabs have been collected each week from up to 15 persons (± 5; any age) presenting with acute respiratory illness (ARI) to each of the 5 selected outpatient health facilities within the KHDSS. The selected facilities are Pingilikani, Mavueni, Mtondia, Matsangoni, and KCRH outpatient department [20] (Fig. 1).
- Homestead community-based surveillance: this is through the respiratory virus reinfections (ResViRe) study that started on August 31, 2023, across 5 administrative locations: Tezo, Roka, Zowerani, Matsangoni, and Ngerenya. Here, participants are visited weekly at home and their respiratory disease status, travel history, and NP/OP swabs are collected from all recruited homestead members regardless of symptom status. If 1 or more homestead member(s) is identified positive for SARS-CoV-2, RSV, or influenza A/B, sample collection frequency for the homestead is increased to twice a week until all members test negative. By March 31, 2024, 64 homesteads (658 participants) had been enrolled and sampled at least once.

For both IP and OP surveillances, samples and relevant participant demographic details (e.g., sex, date of birth, COVID-19 vaccination status, and location/facility) and clinical data were collected once during participant contact with the hospital or health facility where they were recruited [20]. Clinical illness signs and symptoms are recorded by the attending clinician based on a standard questionnaire. The following symptoms were recorded following an interview with the participant or their caregiver: cough, headache, fever (above 37.8 °C), sore throat, vomiting, diarrhoea, difficulty breathing, wheezing, nausea, joint pains, and chest pains.

For the ResViRe study, participant baseline information was captured at recruitment. This included age, sex, vaccinations received, co-morbidities, socioeconomic status, illnesses in the last week, among others. During weekly home visits, a trained fieldworker administered a short questionnaire to collect information about the participant’s current respiratory wellness status, any illness that may have occurred since the last visit, recent travel, and any contact with the healthcare system.

### Laboratory methods

#### Nucleic acid extraction and SARS-CoV-2 screening

Ribonucleic acid (RNA) was extracted from 140 μL NP/OP samples using the automated RNeasy Mini Kit (QIAGEN, UK) following the manufacturer’s protocol. SARS-CoV-2 was screened using an in-house real-time RT-PCR with primers/probe targeting the envelope (E) gene (forward: 5'-ACA GGT ACG TTA ATA GTT AAT AGC GT −3', reverse: 5'-ATA TTG CAG CAG TAC GCA CAC A-3'and probe 5'-ACA CTA GCC ATC CTT ACT GCG CTT CG-3') and QIAGEN Multiplex RT-PCR + R Kit (QIAGEN, UK). Negative and positive controls starting from both the extraction and RT-PCR stages were included in all PCR plates. A sample was considered positive if it had a RT-PCR cycle threshold (Ct) of ≤ 35.0.

#### SARS-CoV-2 whole genome sequencing (WGS)

Positive SARS-CoV-2 samples were subjected to WGS. RNA was re-extracted from 140 µl of sample using the QIAamp Viral RNA Mini Kit (QIAGEN, UK), reverse-transcribed using LunaScript RT SuperMix Kit (New England Biolabs, UK), and the cDNA PCR amplified using Q5 Hot Start High-Fidelity 2 × Mastermix (New England Biolabs, UK) with the ARTIC nCoV-2019 version 5.3.2 primers [25]. Briefly, 8 µl of RNA and 2 µL of LunaScript RT Mix (NEB, E3010, MA, USA) were incubated at 25◦C for 2 min, 55 °C for 10 min, and 95 °C for 10 min and then held at 4 °C. The cDNA was amplified in 2 reaction mixes per sample with the ARTIC nCoV-2019 version 5.3.2 primer pools A and B. The amplification reactions were set up by combining 6.3 μl of Q5 Hot Start High-Fidelity 2X Master Mix (NEB M0494, MA, USA), 1.9 μl of nuclease-free water, 2 μl of primer pool, and 1.3 μl of cDNA. The thermocycling conditions were 1 cycle of 98 °C for 30 s, followed by 25 cycles of 98 °C for 30 s and 65 °C for 5 min, 15 cycles of 62.5 °C for 5 min and 98 °C for 15 s, and 1 cycle of 62.5 °C for 5 min and held at 4 °C indefinitely. The 2 reactions were pooled and cleaned using 1X AMPure XP beads (Beckman Coulter, A63881, Indianapolis, USA) as per the manufacturer’s instructions. The cleaned amplicons were eluted in 20 µl and quantified using a QUBIT fluorometer. Samples with a concentration of > 18 ng/µL were taken forward for library preparation. Sequencing library preparation was undertaken using the COVIDSeq assay (Illumina, USA) and sequenced on the Illumina Miseq platform to generate 100 bp paired-end reads while multiplexing 80 to 100 samples per run.

### Bioinformatic data analysis

#### Genome assemblies and consensus generation

Consensus genomes/sequences were generated by mapping the quality-filtered (using fastp v.0.23.4; *q* ≥ 30 and adapters removed) fastq reads to the Wuhan reference (MN908947.3) using bwa v0.7.17, sorting the mapped reads using samtools v1.10, removing primer sequences, and calling the consensus with a minimum depth of 10 reads using iVar v1.4.2 as previously described [26].

#### Quality control, lineage, and clade assignment

The quality of the newly assembled sequences was assessed using the online Nextclade program version 2.14.1 [27]. Key parameters evaluated included no sequence recovered from the negative control (NC) and the expected sequence for the positive control (PC). Samples yielding complete or near-complete genome sequences (> 70% coverage) were classified into SARS-CoV-2 lineages using the Phylogenetic Assignment of Named Global outbreak Lineages (Pangolin software v1.28) and Nextclade v3.7.1 tools [27]. For the serial samples collected from the same individual in the ResViRe study, only 1 genome was selected per infection episode (see definition below) and included in the downstream analysis based on genome completeness.

#### Global and other Kenya comparison data (“background data”)

This was done per variants with similar secondary level classifications (“PartiallyAlliased”) on Nextclade (i.e., XBB.2.3.*, BA.2.86.1.* (JN.1) and XBB.1.*) (Additional file1: Table S1). The spatial and temporal distribution of the global comparison data is summarized in Additional file 2: Fig. S1. For XBB.2.3-like lineages, all genomic data (*n* = 12,576) on GISAID [6] from October 1, 2023, to June 30, 2024, were downloaded and used for the subsequent phylogenetic analyses. For the JN.1-like lineages, over 500,000 sequences collected between October 1, 2023, to April 30, 2024, were available on GISAID. Metadata for all the sequences was downloaded and subsampled in R to include 5 near-complete sequences per country, month of collection, and lineages identified. This resulted in a total of 5230 sequences used for downstream phylogenetic analysis. For XBB.1-like lineages, 103 XBB.1.34.1 sequences and 187,083 XBB.1.5 sequences were available on GISAID by October 1, 2024. The metadata was downloaded, and the XBB.1.5 sequences were downsampled to include 10 sequences per country and month of collections to give 920 sequences from August 1, 2022, to November 30, 2023. All the 103 XBB.1.34.1 sequences were included.

**Fig. 2:**
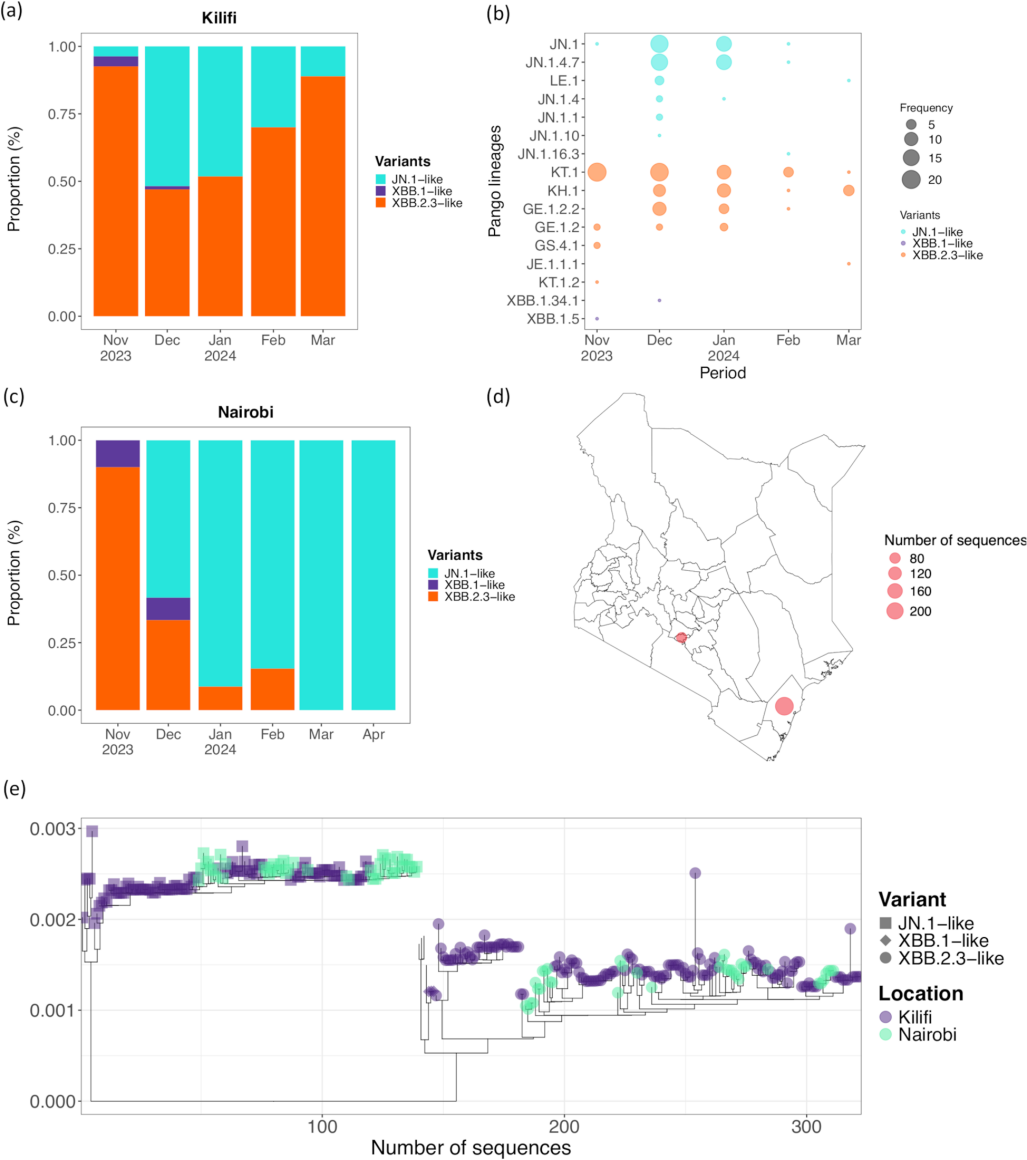
Temporal and spatial distribution of SARS-CoV-2 subvariants/lineages in Kenya, October 2023 to March 2024.** a** Proportionate monthly distribution of 3 major subvariants detected at the Kenyan Coast during the wave. **b** Monthly numbers for the detected 16 Pango lineages during the study period. **c** Proportionate monthly distribution of 3 major subvariants detected in Nairobi during the wave. **d** Map of Kenya showing the 2 counties (Nairobi and Kilifi) with SARS-CoV-2 whole genome data available during the study period. The size of the circles is scaled by their number of SARS-CoV-2 sequences available in GISAID. The map was generated using a shapefile of counties in Kenya from ArcGIS [29]. **e** A time-resolved phylogeny showing the clustering of Kenyan sequences (*n* = 310) collected during the study period. The different tip shapes indicate the different subvariants detected and are colored by the county of sample collection

All Kenyan SARS-CoV-2 genomic data and metadata from January 1, 2023, to October 1, 2024, were downloaded from GISAID and included in the analysis to describe the genomic epidemiology of the virus within the country [28]. The genomic data included 775 sequences collected from 19 counties in Kenya and were classified into 69 Pango lineages. The spatial and temporal distribution of the data is shown in Fig. 2.

All genome sequences and associated metadata in the global dataset are published in GISAID’s EpiCoV database and can be accessed at 10.55876/gis8.241022hw [30]. The EPI_SET_241022hw contains data from 111 countries and territories [30].

#### Pairwise nucleotide differences

Pairwise nucleotide differences from sequence alignments for each lineage were calculated by iterating through all possible sequence pairs using a custom Python script that utilizes Biopython (available on https://github.com/arnoldlambisia/Epiinsights_wave8_Kilifi.git) [31]. Only valid sites, i.e., A, C, T, and G, were considered.

#### Phylogenetic analyses

Multiple sequence alignments (MSA) were generated using Nextalign v1.10.1 (https://.com/neherlab/nextalign) with the Wuhan-Hu-1 SARS-CoV-2 genome (Accession number: NC_045512.2) used as the reference. The alignments were manually checked using AliView v1.27 [32] to determine if any sequences were duplicates or indicative of sequencing/alignment errors and used as input for a maximum likelihood phylogenetic analysis using IQTREE v2.1.3 [33] assuming the general time reversible (GTR) model of nucleotide substitution. Branch support was evaluated with 1000 bootstrap replications. Pairwise nucleotide differences were calculated using *pairsnp* v0.1.0 [34].

### Estimation of molecular clock signal and evolutionary rate

The ML trees were checked for a temporal signal in genetic divergence over time using Tempest v1.5.3 (http://tree.bio.ed.ac.uk/software/tempest/), and outlier sequences that deviated more than 0.0001 from the residual mean were removed. The ML trees were subsequently used to generate time-resolved trees using TreeTime v0.11.1 [35].

Estimation of the time to the most recent common ancestor (TMRCA) and evolutionary rate of the identified variants in Kilifi was performed using the Bayesian approach available in the BEAST software package v1.10.4 [36]. The Kenyan sequences were grouped as JN.1-like (*n* = 87), XBB.2.3-like (*n* = 102, KH.1 removed), and KH.1 (*n* = 35). XBB.1-like were not included due to the small number of sequences. ML trees were generated using IQTREE v2.1.3 (http://www.iqtree.org/) and used to inspect for a temporal clock signal using Tempest v1.5.3 (http://tree.bio.ed.ac.uk/software/tempest/). Time-resolved phylogenies were estimated using the uncorrelated relaxed molecular clock, the HKY model of nucleotide substitution, and the Bayesian skyline demographic model. Analyses were run using 100 million Markov chain Monte Carlo (MCMC) states, sampling every 50,000 steps. Tracer v1.71 [37] was used to check for convergence (effective sample size > 200).

### Phylogeographic analysis

Virus introduction events into Kilifi for each subvariant were inferred from a phylogeographic reconstruction based on the Kenyan genomic sequences data and subsampled background global genomic sequences retrieved from GISAID [30]. These background genomic sequences were selected as described above. Specifically, we conducted a migration analysis as implemented in the TreeTime program, using the country of origin for the global data and Kilifi and non-Kilifi data for Kenyan data as discrete traits to reconstruct geographic locations at all internal nodes. A custom Python script [31]was then used to infer the number of transition events using the annotated tree from the migration analysis by traversing the tree from root to the most external tip and counting every location change and recording the timing of that change [31].

#### Mutational spectrum

To identify evidence of local evolution, nucleotide and amino acid mutations across the sequences were visualized using Snipit v1.4 (https://github.com/aineniamh/snipit) against a local XBB.2.3-like, and JN.1-like reference. The potential fitness effects of the lineage-defining mutations were checked using the https://jbloomlab.github.io/SARS2-mut-fitness/ tool (Additional file 1: TableS1) [38].

### Epidemiological data analyses

#### Definitions

- Symptomatic infection was defined as the occurrence of 1 or more respiratory or systemic infection symptoms (listed in the next section) at sample collection or during the preceding or subsequent week after sample collection for the community cases. The following symptoms were recorded following an interview with the participant or their caregiver: cough, headache, fever (above 37.8 °C), sore throat, vomiting, diarrhoea, difficulty breathing, wheezing, nausea, joint pains, and chest pains.
- Symptomatic fraction was calculated as the proportion of infected individuals who developed/reported having symptoms among the community study (ResViRe) participants.
- An infection episode was defined as a period in which a study participant provided an NP/OP sample that was positive for SARS-CoV-2 by a quantitative RT-PCR (qRT-PCR), sandwiched by a negative RT-PCR test in both the preceding and subsequent 2 weeks.
- An introduction event to Kilifi, Kenya, was defined as a lineage transition event inferred by the migration analysis from any other country or Kenya region outside Kilifi to Kilifi, Kenya.
- A lineage was defined as a SARS-CoV-2 strain designation as described in [39] based on a standard questionnaire.

### Statistical analyses

Temporal trends of SARS-CoV-2 positive cases and lineages were summarized either weekly or monthly. Prevalence was calculated as the number of samples positive divided by the total number of samples collected/tested per platform, and a 95% confidence interval (CI) was given by the Wilson Score method. Kruskal–Wallis and Wilcoxon tests were used to compare median viral load across different variants, age groups, platforms, and symptom status. The distribution of demographic characteristics, variant/lineage proportions, and clinical characteristics was summarized and compared across all the different platforms using a chi-square statistic. Univariate and multivariable logistic regression models were used to examine the association between symptom status (symptomatic vs asymptomatic) and various predictor variables including sex, age (0–4, 5–9, 10–19, 20–39, 40–64, and ≥ 65 years), vaccination status (unvaccinated or vaccinated) and infecting variant (JN.1-like, XBB.2.3-like and XBB.1-like). Results were reported as unadjusted and adjusted odds ratios (ORs) with 95% CIs. *P* values less than 0·05 were considered statistically significant. The data were analyzed using R version 4.1.1 and STATA version 15.0 [40].

## Results

### SARS-CoV-2 infections in the KHDSS—November 2023 to March 2024

The 2023–2024 SARS-CoV-2 wave of infections in the KHDSS began in the second week of November and peaked in the third week of December 2023, after which infection numbers gradually reduced toward the end of March 2024, as evident across the 3 surveillance platforms (Fig. 1). This represents the 8th wave of SARS-CoV-2 in the KHDSS. Between August 31, 2023, and March 31, 2024, a total of 13,847 nasopharyngeal/oropharyngeal (NP/OP) swab samples from 3150 individuals were screened for SARS-CoV-2 by a qRT-PCR targeting the envelope (E) gene. Of these, 308 samples from 235 individuals were found positive (Ct < 35.0). These were collected from 138 individuals enrolled in the community surveillance platform, 88 in the outpatient platform, and 9 in the inpatient platform.

During the peak month of the wave (December 2023), the sample positivity rate was 5.0% (95% CI, 4.0–6.2%) in the community, 15.2% (95% CI, 11.0–20.7%) in outpatients, and 7.6% (95% CI, 2.0–21.9%) in the inpatients (Fig. 1). There were slightly more female positive cases among the identified infections than males (*n* = 136, 56.4%), and the median age among positive cases was 14.0 years (interquartile range 3.0–30.0 years; Table 1). Seven participants enrolled in the community study were suspected of experiencing a repeat infection episode during the outbreak (defined as positive NP/OP samples separated by 14 or more days, with intervening negative tests; Additional file 2: Fig. S2). Among these 7 suspected reinfection cases, 6 were female. Four of the cases involved individuals aged 0 to 1 year, 1 was in the 2- to 4-year age group, 1 was in the 5- to 9-year age group, and 1 was in the 20- to 39-year age group. One case reported having asthma as a comorbidity at the time of recruitment (Table 2).

**Table 1:** Baseline characteristics of SARS-CoV-2 cases identified in 3 KHDSS surveillance platforms between November 2023 and March 2024

	**Community**	**Outpatient**	**Inpatient**	**Total**
Total samples analyzed	11,355	2151	341	13,847
Total participants	658	2151	341	3150
Positive samples	211	88	9	308
Total cases positive	144*	88	9	241
Sequencing status (%)				
Sequenced	115 (79.9)	65 (73.9)	5 (55.6)	185 (76.8)
Sex (%)				
Female	52 (59.1)	2 (22.2)	82 (56.9)	136 (56.4)
Age (years)				
Median (IQR)	12 (3–30.5)	21 (4.7–31.7)	0 (0–0)	14.0 (3.0–30.0)
Age group (%)				
0–4	44 (30.6)	22 (25.0)	9 (100.0)	75 (31.1)
5–9	19 (13.2)	5 (5.7)	-	24 (10.0)
10–19	30 (20.8)	14 (15.9)	-	44 (18.3)
20–39	27 (18.8)	30 (34.1)	-	57 (23.7)
40–64	17 (11.8)	9 (10.2)	-	26 (10.8)
65 +	7 (4.9)	8 (9.1)	-	15 (6.2)
Symptom status (%)				
Asymptomatic	120 (83.3%)	0 (0.0%)	0 (0.0%)	120 (49.8%)
Symptomatic	24 (16.7%)	88 (100.0%)	9 (100.0%)	121 (50.2%)
Vaccination status (%)				
Yes	10 (6.9)	12 (13.6)	-	22 (9.1)
No	134 (93.1)	76 (86.4)	9 (100.0)	219 (90.9)

^*^Note that this includes 7 participants in the community study named respiratory virus reinfections (ResViRe) who had suspected repeat infection episodes

**Table 2:** Demographic, clinical, and laboratory findings for 7 suspected Reinfection Cases from Community Surveillance Study (ResViRe)

Participant (sex)^a^	Age group (years)	Sample date	RT-PCR cycle threshold	Days since previous diagnosis	Infection episode	Clinical status	Lineage	Nt difference^c^	Comment
**Kil/01(F)**	0–1	2023–11-17	20.86	-	1	Asymptomatic	KT.1	-	Probable reinfection
		2023–11-20	30.70	3	1	Asymptomatic	KT.1	0	
		2023–11-23	26.57	3	1	Symptomatic	KT.1	0	
		2023–12-11	31.54	18	2	Asymptomatic	KT.1	4	
**Kil/02(F)**^**b**^	5–9	2024–01-31	23.87	-	1	Asymptomatic	KT.1	-	Probable reinfection
		2024–02-16	34.97	16	2	Asymptomatic	KT.1	29	
**Kil/03(M)**	0–1	2024–01-03	34.66	-	1	Asymptomatic	JN.1	-	Probable reinfection
		2024–01-08	31.02	5	1	Symptomatic	JN.1	2	
		2024–01-11	33.58	3	1	Asymptomatic	JN.1	2	
		2024–02-01	31.38	21	2	Asymptomatic	JN.1	4	
**Kil/04 (F)**	2–4	2023–11-23	27.17	-	1	Asymptomatic	KT.1	-	Probable reinfection
		2023–11-27	28.30	4	1	Asymptomatic	KT.1	0	
		2023–11-30	31.86	3	1	Asymptomatic	KT.1	0	
		2024–03-04	28.17	95	2	Symptomatic	KT.1	17	
		2024–03-07	29.35	3	2	Asymptomatic	KT.1.2	27	
**Kil/05 (F)**	20–39	2023–12-06	34.63	-	1	Asymptomatic	-	-	First episode sample failed sequencing
		2024–01-19	22.98	44	2	Symptomatic	GE.1.2.2		
**Kil/06 (F)**	0–1	2024–01-09	33.66	-	1	Asymptomatic	-	-	Confirmed reinfection
		2024–01-11	33.02	2	1	Asymptomatic	JN.1.4.7	-	
		2024–03-05	22.12	54	2	Asymptomatic	KH.1	101	
		2024–03-08	28.50	3	2	Asymptomatic	KH.1	101	
**Kil/07 (F)**	0–1	2023–12-29	28.60	-	1	Symptomatic	JN.1.4.7	-	Could be a persistent infection
		2024–01-23	30.55	25	2	Asymptomatic	JN.1.4.7	0	

^a^Sex is represented by M for male and F for female

^b^Participant reported having asthma

^c^This refers to the pairwise nucleotide differences from the first sequence identified from the first infection episode

### Clinical presentation and viral load patterns

Cases captured by outpatient (*n* = 88) and inpatient (*n* = 9) surveillance were all symptomatic. Overall, only 9.1% of the cases described here were previously vaccinated (Table 1). Note that in Kenya, COVID-19 vaccination is recommended only for individuals aged 12 years and above, who represent the minority of the infections described in this study. All inpatient cases were discharged alive. The community surveillance collected weekly NP/OP samples regardless of symptom status. For most of the community surveillance positive cases, the infection episodes remained asymptomatic (*n* = 124, 83.2%). Overall, the common symptoms among positive cases were cough (49.2%), fever (above 37.8°C; 27.0%), sore throat (7.3%), headache (6.9%), and difficulty breathing (5.5%). One symptomatic case (< 1 year old) from community surveillance died during an ongoing episode. Among the symptoms observed in this case were coughing, a fever (39.3 °C), a runny nose, and wheezing. The patient was diagnosed with pneumonia and anemia, treated, and discharged. Only 2 out of the 7 suspected reinfection cases identified in the community surveillance were symptomatic (Table 2;Additional file 2: Fig S2). Among the community cases, sex, age, vaccination status, and infecting variant were not associated with the likelihood of being symptomatic during an infection episode (Additional file 1: TableS2).

Using qRT-PCR Ct as a proxy for viral load, there was a statistically significant difference in the approximate viral load at sampling for participants across the 3 sampling platforms. Lower viral loads occurred among the community surveillance samples (Kruskal–Wallis, *p* = 0.0045; Additional file 2: Fig. S3). Furthermore, among cases from the community surveillance, asymptomatic individuals appeared to have a slightly lower viral load (inferred from the higher median Ct value) than the symptomatic individuals, although the difference did not reach statistical significance (Wilcoxon, *p* = 0.33; Additional file 2: Fig. S3). Among the suspected reinfections, the median Ct values were slightly lower for the first infection (28.6; interquartile range (IQR), 25.5–32.0) compared with the reinfection cases (30.5; IQR, 25.6–31.5), with a Wilcoxon test *p *value of 0.9015.

### SARS-CoV-2 lineage variations of the Kilifi 2023–24 wave

Sequencing was performed for at least 1 sample per infection episode for community surveillance and all positive samples from both outpatient and inpatient surveillance platforms. A total of 185 qRT-PCR positive samples from 179 individuals collected between November 14, 2023, and March 27, 2024, in 9 locations of the KHDSS area yielded genomic sequences with > 70% genome coverage; all publicly shared on the GISAID database). The demographic characteristics including age, sex, location or facility, and clinical presentation among sequenced and non-sequenced cases were similar (Additional file 1: TableS3). The median Ct value (inverse viral load) for sequenced cases with ≥ 70% of the genome recovered was significantly lower (*p* < 0.0001; Fig. S2) than the sequenced cases with < 70% of the genome recovered (median, 27.8; IQR, 24.7–30.9) vs. median 32.3 (IQR, 28.7–34.1).

The recovered genomic sequences were classified into 16 Pango lineages within 3 Omicron subvariants—XBB.2.3-like (108 samples), JN.1-like (75 samples), and XBB.1-like (2 samples; Additional file 1: TableS3—with XBB.2.3-like and JN.1-like jointly dominating the wave (98.9% of all cases; Fig. 2). Similar demographic and clinical characteristics were observed in participants infected with JN.1-like, XBB.1-like, and XBB.2.3-like lineages (Additional file 1: TableS4). Seven lineages were identified within XBB.2.3-like cases; KT.1 (*n* = 56), KH.1 (*n* = 25), GE.1.2.2 (*n* = 16), GE.1.2 (*n* = 7), GS.4.1 (*n* = 2), KT.1.2 (*n* = 1), and JE.1.1.1 (*n* = 1; Fig. 2). Equally, 7 lineages were identified within the JN.1-like cases; JN.1 (*n* = 33), JN.1.1 (*n* = 1), JN.1.4 (*n* = 3), JN.1.4.7 (*n* = 30), JN.1.10 (*n* = 1), JN.1.16.3 (*n* = 1), and LE.1 (*n* = 5). Two lineages were identified in XBB.1-like cases: XBB.1.5 (*n* = 1) and XBB.1.34.1 (*n* = 1; Additional file 1: TableS1). Notably, the proportion of XBB.2.3-like virus during the wave was initially high, then declined, and subsequently increased again in Kilifi toward the end of the wave (Fig. 2). Genomic sequencing and typing revealed that earlier the virus belonged to the GE.1.2.1 and KT.1 lineages within the subvariant, whereas later it was identified as the genetically distinct KH.1 lineage.

**Fig. 3:**
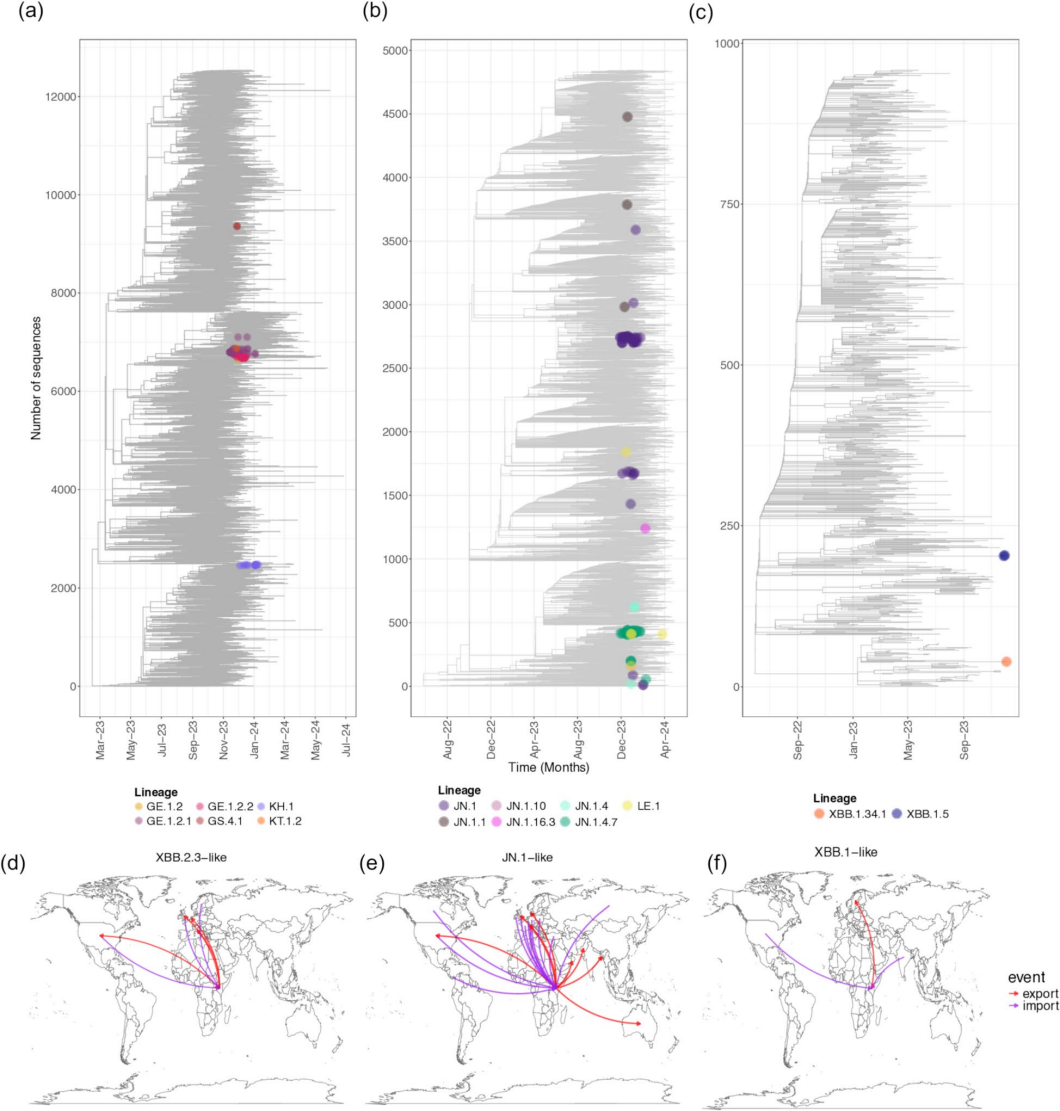
Time-resolved phylogenetic trees for XBB.2.3-like (**a**), JN.1-like (**b**), and XBB.1-like (**c**) lineages, including sequences sampled on a global basis. The tip points are Kilifi sequences colored by the Pangolin lineages and the noncolored tips are global sequences for context. Global patterns of introduction and exportation events across the observed **d** XBB.2.3-like, **e** JN.1-like, and **f** XBB.1-likevariants using time-resolved phylogenies. The arrows move from country of origin to destination are colored by the importation (purple) and exportation events (red) to and from coastal Kenya. The maps were generated using the sp package in *R* [42]

The median Ct values did not differ across the different variants detected (Kruskal–Wallis, *p* = 0.53) or by participant age groups (Kruskal–Wallis, *p* = 0.35; Additional file 1:FigS3). Based on the community surveillance platform, there was no difference in symptom status across the 3 subvariants (JN.1-like, XBB.2.3-like, and XBB.1-like) infected participants (*p* = 0.31). Pairs of first and subsequent episode viruses were sequenced in 6 of the 7 suspect infection cases. Notably, in all but one, the lineage in the subsequent virus was found to be the same, although multiple nucleotide differences were detected (see below). Samples from both the first and second episodes recovered the KT.1 sequence in 3 participants, JN.1 in 1 participant, and JN.1.4.7 in 1 participant. Only a single participant exhibited a different lineage in the second episode, where the first infection was with JN.1.4.7 and the second was KH.1 (Table 2).

### Divergence of the detected XBB.2.3-like and JN.1-lineages

Most (74.1%) of the XBB.2.3-like detections in the 2023/24 KHDSS wave had the GE.1.2 lineage (alias XBB.2.3.10.1.2) backbone, which is characterized by the 2 amino acid changes in the spike (S) protein: A376S and S:X478T. Three descendants of this lineage were observed: KT.1 (S: K77R), KT.1.2 (ORF1b: N232S), and GE.1.2.2 (S: S408N, ORF1b: A517V, ORF1b: D1899E, ORF7a-ORF8 completed deleted). These were detected in both the community-based and outpatient surveillance platforms. Globally, based on GISAID data, the GE.1.2 lineage was first detected in the USA (February 4, 2023) [6]. Detections in Kenya started in May 2023, mostly in Nairobi (Fig. 2) [41]. The first detection of GE.1.2 in Kilifi was in November 2023, although this virus had acquired additional nonsynonymous (ORF1a: Q1519H, ORF3a: L52F, ORF3a: W128C, and ORF6: D61H) and synonymous mutations (T2506C, T25345C, 26340 T; Additional file 2: Fig. S4). The Kilifi KH.1 genomes clustered separately from the rest of the available global KH.1 sequences (Additional file 2: Fig. S5). Within the XBB.2.3-like lineages, there was varied within-lineage genetic diversity the median pairwise nucleotide differences ranging from 7 to 22 (Additional file 2: Fig. S6). The KH.1 lineage showed a mean evolutionary rate of 4.5 × 10^−4^ nucleotide substitutions/site/year (95% highest posterior density (HPD) 1.3 × 10^−4^ to 8.2 × 10^−4^), whereas the evolutionary rate in the other XBB.2.3-like lineages was 8.0 × 10^−4^ (95% HPD 5.1 × 10^−4^ to 1.0 × 10^−3^).

Most of our JN.1-like detections had the JN.1 lineage (alias BA.2.86.1.1) backbone characterized by amino acid changes S: L455S, ORF1a: R3821K, and ORF7b: F19L. Six descendants of this lineage were observed: JN.1.1 (ORF1a: F499L), JN.1.4 (ORF1a: T170I), JN.1.4.7 (ORF3a: G18D), LE.1 (S: R346T), JN.1.10 (S: T95I), and JN.1.16.3 (S: T572I). Within the JN.1.4.7 lineage, we observed clustering per geographic region among the Kenya sequences (Additional file 1: Fig. S5). Two of the JN.1.4.7 sequences from Kilifi had an ORF1a: T1881I mutation that was lacking in the Nairobi sequences. We observed within-lineage genetic diversity among the JN.1-like lineages with a median pairwise nucleotide difference ranging from 3 in JN.1.4.7 to 44 in JN.1.4 (Additional file 2: Fig S6). The estimated evolutionary rate for the JN.1-like variants was 9.8 × 10^−4^ nucleotide substitutions/site/year (95% HPD 5.6 × 10^−4^ to 1.3 × 10^−3^). Sequences generated from the 3 platforms clustered together in lineage-specific phylogenetic trees, indicating genetically similar viruses across all outpatient, inpatient, and community-based surveillance.

### Sequence relatedness in suspected reinfection cases

The sequence data from the suspected reinfection cases were compared through pairwise nucleotide comparisons to the earliest sequenced sample from each reinfected individual (Additional file 2: Fig. S2). Due to the age cutoff for COVID-19 vaccination in Kenya (over 12 years), all suspected reinfection cases listed here were ineligible to receive the COVID-19 vaccine, with the exception of 1 individual aged between 20 and 29 years (for which the vaccine information was missing). As expected, the individual infected by 2 different Omicron subvariants (JN.1.4.7 then KH.1) exhibited the highest pairwise genetic distance (88 nt). Also, samples collected within a 2-week period (< 14 days) consistently showed minimal nucleotide differences (< 3 nt). In contrast, all except 1 of the individuals who tested positive again after the 2-week window displayed considerable genetic variation in the later samples, more consistent with a reinfection (Table 2). Notably, in 4 participants who appeared reinfected by the same Omicron lineage, samples collected after the 2-week period exhibited nucleotide differences of 4 nt, 17 nt, 27 nt, and 29 nt, indicating probable reinfections with strains within the same Pango lineage. The 1 exception was a participant (0.68 years old) who appeared to have been reinfected 25 days later with a genetically identical virus as the first infection (Additional file 2: Fig. S2).

### Dynamics of virus introduction and exportation events to and from KHDSS

Using the migration model in TreeTime [35], we inferred patterns of virus introduction and exportation events into and out of the Kilifi HDSS area. We first reconstructed time-resolved maximum likelihood phylogenies for the XBB.1-like, XBB.2.3-like, and JN.1-like lineages based on the 185 newly sequenced Kilifi SARS-CoV-2 genomes and 18,728 “background” genomes from other countries and the Nairobi region in Kenya (Fig. 3). From these time-resolved phylogenies, we estimated that there were at least 38 SARS-CoV-2 introduction events into Kilifi, 22 for JN.1-like, 14 for XBB.2.3-like, and 2 for XBB.1-like. The introductions were predominantly from North America (*n* = 19), Europe (*n* = 18), and Nairobi-Kenya (*n* = 1). We also identified 18 exportation events from Kilifi (a) to other regions in Kenya (*n* = 4) and (b) to countries in Europe (*n* = 7), Asia (*n* = 3), North America (*n* = 2), Oceania (*n* = 1), and Africa (*n* = 1). Of these export events, 9 involved JN.1-like lineages while 6 involved XBB.2.3-like lineages (Fig. 3).

## Discussion

Despite the absence of adequate continued SARS-CoV-2 genomic surveillance in LMICs, our study team has maintained genomic surveillance, enabling us to report the epidemiology and viral genomic dynamics of the SARS-CoV-2 wave between November 2023 and March 2024 in the KHDSS area of Kilifi, Kenya. Genomic analysis of over 180 samples revealed that the wave was dominated by the GE.1.2. * lineage, an XBB.2.3-like subvariant, and JN.1-like lineages that arrived through direct or indirect multiple virus introductions from outside Kenya. Limited cases of XBB.1.5 and XBB.1.34 were identified, but did not appear to spread widely. Our analyses provide the first detailed description of the introduction and further dissemination of JN.1* and XBB.2.3* subvariants in Kenya.

The XBB.2.3* strains are a descendant of the recombinant XBB variant [43], and XBB evolution resulted in mutations enhancing its immune escape relative to earlier Omicron lineages [44, 45]. Among these is XBB.2.3* (characterized by S: D253G and S: P521S) that we report here, and which emerged later in 2022 [46]. Available data on public sequence databases (e.g., GISAID [6]) suggests that XBB.2.3* spread worldwide. Its prevalence in the USA in May 2023 was ~ 3.2% [47]. The earliest XBB.2.3* available from Nairobi, Kenya, was collected in May 2023, but the strain was not detected in Kilifi until November 2023.

The JN.1* lineages are descendants of BA.2.86 Omicron subvariant (first identified July 2023) [48]. Approximately 62% of the SARS-CoV-2 sequences deposited on the GISAID database during our study period were JN.1-like, indicating this subvariant’s global predominance [6]. The epidemiological success of JN.1* is hypothesized to be in part due to its additional spike amino acid change (L455S) that facilitates antibody escape, greater ACE-2 binding affinity, and increased infectivity [48, 49]. However, unlike most regions globally where JN.1-like lineages were predominant in late 2023/early 2024, XBB.2.3-like lineages were dominant in coastal Kenya, comprising 58.3% (95% CI, 50.9–65.4%) of our sequenced samples.

During our study period, several XBB.1.9 descendants (EG.5, HK.3, and HV.1) were reported globally, albeit at low frequency (1–11%) [6]. These were not detected in our surveillance. Only a single case of XBB.1.5 (S: G252V and S: F486P) was detected during the Kenyan wave, although this lineage had predominated infections in some global locations including the USA [50]. In early 2023, the FY.4 lineage dominated in the KHDSS area and is thought to have emerged in-country [14, 51]. Overall, therefore, it appears that the dominant virus lineages in Kilifi during new waves of infection are not always aligned with those that dominate globally. This may reflect stochastic founder events or a unique local SARS-CoV-2 immunological landscape that plays a role in selecting locally predominant variants.

In this study, ~ 83% of the infected individuals remained asymptomatic based on our community-based surveillance that tests individuals regardless of symptom status. This aligns with our previous findings in the region: 79% (95% CI, 66–92%) asymptomatic infection among secondary household infection cases [52] and 92.4% among individuals who received a test for various reasons early in the pandemic [53]. The high asymptomatic infection prevalence in many African settings has been hypothesized to be potentially due to local climate, human genetics, cross-reacting immunity from endemic infections, and potential underreporting of symptoms [54]. Other regions of the world have reported lower asymptomatic infection rates e.g., ~ 6–47% in China and the USA among SARS-COV-2 cases during the Omicron waves [55, 56].

By October 2023, coastal Kenya had experienced up to 7 waves of SARS-CoV-2 infections [10–13, 26]. A year earlier (November 2022), SARS-CoV-2 seroprevalence in the region was estimated at 77.4% [8] comparable with other regions globally where seroprevalence was estimated to be 50–98% during the same period [57–61]. This high seroprevalence is hypothesized to primarily result from natural infections, as only about 27% of Kenyan adults had received COVID-19 vaccines [62]. Most of the infections in the wave we describe here were in children (median age 14 years). Adult infections during this COVID-19 wave were likely lower because most adults have some level of immunity from past exposure or vaccination. Notably, Kenya has not recommended COVID-19 vaccinations for children aged under 12 years. Further investigation into the COVID-19 burden among children and the benefits of vaccinating this frequently immunologically naive group is essential.

Omicron variant infections have been associated with milder symptoms compared with earlier variants [1, 4, 5, 63]. Data on clinical differences among the emergent Omicron lineages are currently limited. A study from Singapore found no significant difference in hospitalization risk among XBB variants but noted a higher risk of severe outcomes associated with XBB.1.16 [64]. We found similar clinical characteristics across Omicron subvariants XBB.2.3-, JN.1-, and XBB.1-like. Notably, individuals attending outpatient clinics commonly presented with upper respiratory symptoms (cough and sore throat) and non-respiratory symptoms (fever and headaches), like what was experienced with earlier Omicron lineages [65].

While SARS-CoV-2 reinfections have been widely documented globally, particularly following the emergence of the Omicron variant [66] data on these cases in sub-Saharan Africa remain limited [18]. This study is the first to compare genetic sequences from suspected reinfections in the region, providing crucial genomic evidence of repeat infections, some occurring within remarkably short intervals of less than 3 weeks, with minimal genetic variation among the viruses. Although uncommon, we show that individuals can experience multiple SARS-CoV-2 episodes within the same wave. One study in Spain found that 42% of 26 patients with COVID-19 episodes separated by 20–45 days had reinfections with different variants [67]. Interestingly, these early reinfections showed no specific clinical patterns, occurring predominantly among unvaccinated individuals and those under 18 years of age [67]. The mechanisms underlying these short-interval reinfections are not fully understood, and immunological characterization of the reinfected participants in our community-based surveillance cohort is currently ongoing.

This study had several limitations. First, genome sequencing of positive samples failed in 23.2% of the samples, largely due to low viral loads. This may impact the lineage proportions observed if some failures were associated with the genetic characteristics of the infecting lineage. Second, the health facility (HF) surveillance platform was interrupted for 2 weeks (Christmas and New Year’s Eve weeks). This interruption period unfortunately coincided with the peak weeks for the epidemic as inferred from the community surveillance. This resulted in missing data on the lineage distribution for the outpatient surveillance at the epidemic peak and clinical presentation. Third, our surveillance is focused on the KHDSS area (~ 900 km[2]), with the community surveillance undertaken in the northern area alone. As much as this might be representative of Kilifi, it may be less so for the rest of Kenya. Inclusion of publicly available sequence data from Nairobi identified 7 lineages not detected in Kilifi. There is a need for a systematic countrywide surveillance platform.

## Conclusions

In conclusion, we provide insights into the molecular epidemiology of a SARS-CoV-2 infection wave that occurred from November 2023 to March 2024 in coastal Kenya, during a post-pandemic wave. This is the first detailed description of JN.1 lineage introduction into Kenya and its transmission and evolution in a sub-Saharan Africa setting [41, 68–71]. We found that this (8th) wave of SARS-CoV-2 infections in coastal Kenya was comprised of multiple co-circulating lineages within 3 subvariants (JN.1*, XBB.2.3*, and XBB.1*), which were seeded several times into the local population. Unlike JN.1, which was most likely first introduced into Kenya shortly before detection in Kilifi, XBB.2.3* lineages were in circulation elsewhere in Kenya since early 2023, although we also observed important genetic differences in the Kilifi strains from those earlier detected elsewhere in the country. As was the case for previous waves, most of the SARS-CoV-2 infections during this new wave (> 80%) were asymptomatic, although there were still some instances of severe disease. We recommend further studies to illuminate the factors responsible for this. This 8th wave of infections in coastal Kenya provides more evidence that SARS-CoV-2 continues to transmit and evolve in humans, causing periodic outbreaks in communities. Sustained genomic surveillance informs us of any changes in the local epidemiology of the virus as well as the origins of new waves, allowing optimisation of available countermeasures.

## Supplementary Information

Supplementary Material 1: Table S1: Description of PANGO lineages identified in Kilifi, 2023–24. Table S2: Determinants of SARS-CoV-2 symptom status among community cases in coastal Kenya. Table S3: Comparison of demographic and clinical characteristics between sequenced and not sequenced samples. Table S4: Comparison of demographic characteristics and clinical presentation across SARS-CoV-2 variants among cases in Kilifi.

Supplementary Material 2: Fig. S1: Spatial and temporal distribution of sampled SARS-CoV-2 sequence from GISAID for XBB.2.3-like, JN.1-like and XBB.1-like variants. Fig. S2: Suspected reinfections in the community-based surveillance and genomic findings. Fig. S3: Patterns of SARS-CoV-2 cycle threshold (indicative and inverse to virus load quantities). Fig. S4: Amino acid differences in the ORF1a, ORF3a and ORF6 among lineage GE.1.2 sequences (n = 62) collected in multiple locations across Kenya. Fig. S5: Maximum likelihood trees for 5 lineages commonly detected lineages. Fig. S6: Genetic diversity within identified lineages in Kenya between October 2023 and April 2024.

## Data Availability

The final consensus genomes from the SARS-CoV-2 samples sequenced in this study have been deposited in the Global Initiative on Sharing all Influenza Data (GISAID) database (10.55876/gis8.250116pz) [28]. Epidemiological data and scripts for data analysis are available on the Harvard Dataverse (10.7910/DVN/BMCJTI)[72].

## Abbreviations

SARS-CoV-2: Severe acute respiratory syndrome coronavirus 2
KHDSS: Kilifi Health and Demographic Surveillance
C.I: Confidence interval
COVID-19: Coronavirus disease 2019
VOC: Variants of concern
SNPs: Single nucleotide polymorphisms
WHO: World Health Organisation
PHEIC: Public health emergency of international concern status
LMICs: Low- and middle-income country settings
KWTRP: KEMRI-Wellcome Trust Research Programme
PEO: Pathogen Epidemiology and Omics Group
KCRH: Kilifi County Referral Hospital
IP: Paediatric inpatient
OP: Outpatient
ResViRe: Respiratory virus reinfections
WGS: Whole genome sequencing
Pangolin: Phylogenetic Assignment of Named Global outbreak Lineages ()
TMRCA: Time to the most recent common ancestor
MCMC: Markov chain Monte Carlo
qRT-PCR: Quantitative real-time reverse transcription polymerase chain reaction
ORs: odds ratios
IQR: interquartile range
GISAID: Global Initiative on Sharing All Influenza Data
HPD: Highest posterior density
SERU: Scientific Ethics and Research Unit
