# Supplementary material for: Genomic and clinical epidemiology of SARS-CoV-2 in coastal Kenya: insights into variant circulation, reinfection, and multiple lineage importations during a post-pandemic wave
Source: BMC Glob Public Health. 2025 Sep 9;3:80. doi: 10.1186/s44263-025-00201-6 (PMC12421758; doi:10.1186/s44263-025-00201-6)
Supplement: Supplementary file 1 — Supplementary Material 1: Table S1: Description of PANGO lineages identified in Kilifi, 2023–24. Table S2: Determinants of SARS-CoV-2 symptom status among community cases in coastal Kenya. Table S3: Comparison of demographic and clinical characteristics between sequenced and not sequenced samples. Table S4: Comparison of demographic characteristics and clinical presentation across SARS-CoV-2 variants among cases in Kilifi. [file 44263_2025_201_MOESM1_ESM.docx]

## **Table S1:** Description of PANGO lineages identified in Kilifi, 2023-24.

| Pango lineage | Alias | Frequency (%; *n* = 185) | Lineage defining mutations | Comment (known Impact of changes) |
| --- | --- | --- | --- | --- |
| **JN.1-like** |  | **75 (31.1%)** | **S: L455S, ORF1a: R3821K, ORF7b: F19L** | Increased neutralization resistance ^1,2^ |
| JN.1 | BA.2.86.1.1 | 33 (13.7) | S: L455S, ORF1a: R3821K, ORF7b: F19 |  |
| JN.1.4.7 | BA.2.86.1.1.4.7 | 30 (12.4) | ORF3a: G18D |  |
| LE.1 | BA.2.86.1.1.4.7.1 | 5 (2.1) | S: R346T |  |
| JN.1.4 | BA.2.86.1.1.4 | 3 (1.2) | ORF1a: T170I |  |
| JN.1.1 | BA.2.86.1.1.1 | 2 (0.8) | ORF1a: F499L, C11747T |  |
| JN.1.10 | BA.2.86.1.1.10 | 1 (0.4) | S: T95I |  |
| JN.1.16.3 | BA.2.86.1.1.16.3 | 1 (0.4) | S: T572I |  |
| **XBB.2.3-like** |  | **108 (44.8)** | **Defined by S: P521S and S: S486P** |  |
| KT.1 | XBB.2.3.10.1.2.1.1 | 56 (23.2) | S: K77R |  |
| KH.1 | XBB.2.3.3.1.2.1.1.1.1.1 | 25 (10.4) | S: E554K, on N: L13F, ORF1a: R135S |  |
| GE.1.2.2 | XBB.2.3.10.1.2.2 | 16 (6.6) | S: S408N, ORF1b: A517V and ORF1b: D1899E | ORFa-ORF8 possibly completely deleted |
| GE.1.2 | XBB.2.3.10.1.2 | 7 (2.9) | S: N148T | Kenya lineage |
| GS.4.1 | XBB.2.3.11.4.1 | 2 (0.8) | S: N185D and ORF3a:G172C |  |
| JE.1.1.1 | XBB.2.3.3.1.2.1.1.1.1 | 1 (0.4) | S: Q52R |  |
| KT.1.2 | XBB.2.3.10.1.2.1.1.2 | 1 (0.4) | ORF1b: N2328S, C583T |  |
| **XBB.1-like** |  | **2 (0.8)** | **Defined by S: G252V** | Low fitness change ^2^ |
| XBB.1.34.1 |  | 1 (0.4) | S: E554K, ORF1b: P970 |  |
| XBB.1.5 |  | 1 (0.4) | S: F486P |  |
| Description based on information provided in https://github.com/cov-lineages/pango-designation/blob/master/lineage_notes.txt Note that two lineages are thought to have arisen in Kenya | | | | |

## **Table S2**: Determinants of SARS-CoV-2 symptom status among community cases in coastal Kenya

|  | Unadjusted OR | P value | Adjusted OR | *p* value |
| --- | --- | --- | --- | --- |
| Sex^1^ |  |  |  |  |
| Male | 0.45 (0.15 - 1.21) | 0.01 | 0.38 (0.11 - 1.17) | 0.11 |
| Age group^2^ |  |  |  |  |
| 5-9 | 0.45 (0.06 - 2.02) | 0.3 | 0.26 (0.01 - 2.02) | 0.3 |
| 10-19 | 0.35 (0.05 - 1.53) | 0.2 | 0.22 (0.01 - 1.39) | 0.2 |
| 20-39 | 1.16 (0.34 -3.72) | 0.7 | 1.37 (0.35 -5.05) | 0.6 |
| 40-64 | 0.97 (0.26 - 3.22) | 0.9 | 1.50 (0.37 - 5.65) | 0.5 |
| 65+ | 7.57e-08 (NA - 1.54e+72) | 0.9 | 4.07e-08 (NA - 3.63e+123) | 0.9 |
| Vaccination Status^3^ |  |  |  |  |
| Vaccinated | 1.06 e-07 (NA - 5.81e+34) | 1.90E-11 | 2.17e-08 (NA - 2.91e+102) | 0.9 |
| Variant^4^ |  |  |  |  |
| XBB.2.3-like | 0.68 (0.26 - 1.78) | 0.4 | 0.61 (0.21 - 1.72) | 0.3 |
| XBB.1-like | 2.0 e+7 (3.5e-122 - NA) | 0.9 | 4.01e+8 (0.00 - NA) | 0.9 |
| ^1^Reference group is female  ^2^Reference group is 0-4  ^3^Reference group is unvaccinated  ^4^Reference group is JN.1-like | | | | |

## **Table S3:** Comparison of demographic and clinical characteristics between sequenced and not sequenced samples.

|  | Sequenced (*n* = 185) | Not sequenced (*n* = 56) | Total (*n* = 241) | *p* value |
| --- | --- | --- | --- | --- |
| **Platform (%)** |  |  |  | 0.177 |
| Community | 115 (62.2) | 29 (51.8) | 144 (59.8) |  |
| Outpatient | 65 (35.1) | 23 (41.1) | 88 (36.5) |  |
| Inpatient | 5 (2.7) | 4 (7.1) | 9 (3.7) |  |
| **Sex (%)** |  |  |  | 0.424 |
| Female | 107 (57.8) | 29 (51.8) | 136 (56.4) |  |
| **Age (Years)** |  |  |  | 0.216 |
| Median (range) | 13 (0.0 - 104.0) | 14 (0.0 - 65.0) | 14 (0.0 - 104.0) |  |
| **Age group (%)** |  |  |  | 0.653 |
| 0-4 | 58 (31.4) | 17 (30.4) | 75 (31.1) |  |
| 5-9 | 18 (9.7) | 6 (10.7) | 24 (10.0) |  |
| 10-19 | 32 (17.3) | 12 (21.4) | 44 (18.3) |  |
| 20-39 | 42 (22.7) | 15 (26.8) | 57 (23.7) |  |
| 40-64 | 21 (11.4) | 5 (8.9) | 26 (10.8) |  |
| 65+ | 14 (7.6) | 1 (1.8) | 15 (6.2) |  |
| **Symptom status (%)** |  |  |  | 0.787 |
| Asymptomatic | 93 (50.3) | 27 (48.2) | 120 (49.8) |  |
| Symptomatic | 92 (49.7) | 29 (51.8) | 121 (50.2) |  |
| **Clinical characteristics (%)** |  |  |  | 0.932 |
| Coughing | 88 (47.6) | 27 (48.2) | 115 (47.7) |  |
| Diarrhea | 3 (1.6) | 0 (0.0) | 3 (1.2) | 0.338 |
| Headache | 15 (8.3) | 1 (1.9) | 16 (6.9) | 0.108 |
| Fever | 44 (23.8) | 15 (26.8) | 59 (24.5) | 0.647 |
| Vomiting | 4 (2.2) | 1 (1.8) | 5 (2.1) | 0.863 |
| Sore throat | 11 (6.1) | 3 (5.8) | 14 (6.0) | 0.927 |
| Chest pains | 5 (2.8) | 1 (1.9) | 6 (2.6) | 0.732 |
| Difficulty breathing | 7 (3.8) | 7 (12.5) | 14 (5.8) | 0.015 |
| Joint pains | 4 (2.2) | 0 (0.0) | 4 (1.7) | 0.278 |
| Wheezing | 2 (1.1) | 1 (1.8) | 3 (1.2) | 0.677 |

## **Table S4:** Comparison of demographic characteristics and clinical presentation across SARS-CoV-2 variants among cases in Kilifi.

|  | JN.1-like (%; *n* = 75) | XBB.1-like (%; *n* = 2) | XBB.2.3-like (%; *n* = 108) | Total (%; *n* = 185) | p value |
| --- | --- | --- | --- | --- | --- |
| **Sex** |  |  |  |  | 0.171 |
| Female | 41 (54.7) | 0 (0.0) | 66 (61.1) | 107 (57.8) |  |
| **Age group in years** |  |  |  |  | 0.973 |
| 0-4 | 23 (31.1) | 1 (50.0) | 34 (31.5) | 58 (31.5) |  |
| 5-9 | 8 (10.8) | 0 (0.0) | 10 (9.3) | 18 (9.8) |  |
| 10-19 | 11 (14.9) | 0 (0.0) | 21 (19.4) | 32 (17.4) |  |
| 20-39 | 17 (23.0) | 1 (50.0) | 24 (22.2) | 42 (22.8) |  |
| 40-64 | 10 (13.5) | 0 (0.0) | 10 (9.3) | 20 (10.9) |  |
| 65+ | 5 (6.8) | 0 (0.0) | 9 (8.3) | 14 (7.6) |  |
| Missing data | 1 | 0 | 0 | 1 |  |
| **Symptom status** |  |  |  |  | 0.307 |
| Asymptomatic | 40 (53.3) | 0 (0.0) | 53 (49.1) | 93 (50.3) |  |
| Symptomatic | 35 (46.7) | 2 (100.0) | 55 (50.9) | 92 (49.7) |  |
| **Clinical presentation** |  |  |  |  |  |
| Coughing | 35 (46.7) | 2 (100.0) | 51 (47.2) | 88 (47.6) | 0.327 |
| Fever | 16 (21.3) | 1 (50.0) | 27 (25.0) | 44 (23.8) | 0.578 |
| Headache | 6 (8.2) | 0 (0.0) | 9 (8.6) | 15 (8.3) | 0.909 |
| Sore throat | 5 (6.8) | 0 (0.0) | 6 (5.7) | 11 (6.1) | 0.892 |
| Difficulty breathing | 3 (4.0) | 0 (0.0) | 4 (3.7) | 7 (3.8) | 0.956 |
| Chest pains | 3 (4.1) | 0 (0.0) | 2 (1.9) | 5 (2.8) | 0.659 |
| Vomiting | 3 (4.0) | 0 (0.0) | 1 (0.9) | 4 (2.2) | 0.364 |
| Joint pains | 2 (2.7) | 0 (0.0) | 2 (1.9) | 4 (2.2) | 0.912 |
| Diarrhoea | 2 (2.7) | 0 (0.0) | 1 (0.9) | 3 (1.6) | 0.646 |
| Wheezing | 1 (1.3) | 0 (0.0) | 1 (0.9) | 2 (1.1) | 0.956 |
