# Supplementary material for: Genomic and clinical epidemiology of SARS-CoV-2 in coastal Kenya: insights into variant circulation, reinfection, and multiple lineage importations during a post-pandemic wave
Source: BMC Glob Public Health. 2025 Sep 9;3:80. doi: 10.1186/s44263-025-00201-6 (PMC12421758; doi:10.1186/s44263-025-00201-6)
Supplement: Supplementary file 2 — Supplementary Material 2: Fig. S1: Spatial and temporal distribution of sampled SARS-CoV-2 sequence from GISAID for XBB.2.3-like, JN.1-like and XBB.1-like variants. Fig. S2: Suspected reinfections in the community-based surveillance and genomic findings. Fig. S3: Patterns of SARS-CoV-2 cycle threshold (indicative and inverse to virus load quantities). Fig. S4: Amino acid differences in the ORF1a, ORF3a and ORF6 among lineage GE.1.2 sequences (n = 62) collected in multiple locations across Kenya. Fig. S5: Maximum likelihood trees for 5 lineages commonly detected lineages. Fig. S6: Genetic diversity within identified lineages in Kenya between October 2023 and April 2024. [file 44263_2025_201_MOESM2_ESM.docx]

**Fig. S1**: Spatial and temporal distribution of sampled SARS-CoV-2 sequence from GISAID for XBB.2.3-like, JN.1-like and XBB.1-like variants. World maps showing the origin of subsampled sequences with the size of the circles corresponding to the number of sequences for **(a)** XBB.2.3-like, **(b)** JN.1-like and **(c)** XBB.1-like. The maps were generated using the sp package in R ^1^. Temporal distribution (monthly) of the subsampled sequences for **(d)** XBB.2.3-like, **(e)** JN.1-like and **(f)** XBB.1-like subvariants

**Fig. S2**: Suspected reinfections in the community-based surveillance and genomic findings. **(A)** NP/OP temporal sampling patterns in the seven participants with suspected reinfection events The-axis labels show participant number and in brackets their sex (M for male and F for female) followed by their age in years**. (B)** Each panel is a sequence alignment for positive samples from six of the seven suspected reinfection cases. The genomes are compared against the earliest positive sample from the individual (bottom line of each graph) except in Kil/03 where the second sequence was used due to many gaps observed in the initial sample. Colored bars indicate single nucleotide changes from the initial patient sequence with the color coding orangered, slateblue, crimson and indigo and for A, C, T and G respectively.

**Fig. S3**: Patterns of SARS-CoV-2 cycle threshold (indicative and inverse to virus load quantities). Comparison across different genome completeness (**A**) surveillance platforms (**B**), clinical presentation (**C**; only for community surveillance), sub-variants (**D**) and age groups (**E**) in samples collected between November 2023 and March 2024 from coastal Kenya.

**Fig. S4**: Amino acid differences in the ORF1a, ORF3a and ORF6 among lineage GE.1.2 sequences (n = 62) collected in multiple locations across Kenya. The changes highlighted in a rectangular box are mutations that were seen mainly in Kilifi sequences.

**Fig. S5**: Maximum likelihood trees for five lineages commonly detected lineages. The phylogenies show the evolutionary clustering of Kenyan and global sequences of the lineages collected between October 2023 and June 2024. Global data for contextual purposes are shown as non-colored tips and Kenyan sequences are colored The tips are shaped by location in Kenya either Nairobi or Kilifi.

.

**Fig. S6**: Genetic diversity within identified lineages in Kenya between October 2023 and April 2024. The bar plots show pairwise nucleotide differences between all possible sequence pairs within a lineage. The red dotted line shows the median pairwise nucleotide difference within the lineage.

References:

1. Pebesma EJ, Bivand R. Classes and methods for spatial data in R. R News [Internet]. 2005 Nov;5(2):9–13. Available from: https://CRAN.R-project.org/doc/Rnews/
